# A calmodulin inhibitor, W-7 influences the effect of cyclic adenosine 3', 5'-monophosphate signaling on ligninolytic enzyme gene expression in *Phanerochaete chrysosporium*

**DOI:** 10.1186/2191-0855-2-7

**Published:** 2012-01-24

**Authors:** Takaiku Sakamoto, Yuki Yao, Yoshifumi Hida, Yoichi Honda, Takashi Watanabe, Wataru Hashigaya, Kazumi Suzuki, Toshikazu Irie

**Affiliations:** 1 Environmental Science Graduate School, The University of Shiga Prefecture, 2500 Hassaka-cho, Hikone City, Shiga, 522-8533, Japan; 2 Research Institute for Sustainable Humanosphere, Kyoto University, Gokasho, Uji, Kyoto, 611-0011, Japan

**Keywords:** *Phanerochaete chrysosporium*, cAMP signaling, Calmodulin signaling, Lignin peroxidase, Manganese peroxidase

## Abstract

The capacity of white-rot fungi to degrade wood lignin may be highly applicable to the development of novel bioreactor systems, but the mechanisms underlying this function are not yet fully understood. Lignin peroxidase (LiP) and manganese peroxidase (MnP), which are thought to be very important for the ligninolytic property, demonstrated increased activity in *Phanerochaete chrysosporium *RP-78 (FGSC #9002, ATCC MYA-4764™) cultures following exposure to 5 mM cyclic adenosine 3', 5'-monophosphate (cAMP) and 500 μM 3'-isobutyl-1-methylxanthine (IBMX), a phosphodiesterase inhibitor. Real-time reverse transcription polymerase chain reaction (RT-PCR) analysis revealed that transcription of most LiP and MnP isozyme genes was statistically significantly upregulated in the presence of the cAMP and IBMX compared to the untreated condition. However, 100 μM calmodulin (CaM) inhibitor N-(6-aminohexyl)-5-chloro-1-naphthalenesulfonamide (W-7), which had insignificant effects on fungal growth and intracellular cAMP concentration, not only offset the increased activity and transcription induced by the drugs, but also decreased them to below basal levels. Like the isozyme genes, transcription of the CaM gene (*cam*) was also upregulated by cAMP and IBMX. These results suggest that cAMP signaling functions to increase the transcription of LiP and MnP through the induction of *cam *transcription.

## Introduction

White-rot fungi are known to have a powerful ligninolytic system that can completely degrade wood lignin (Kirk and Farrell 1987; Kirk et al. 1975) as well as persistent organic pollutants such as dioxin (Bumpus et al. 1985). This ability may be applicable to the construction of a novel potent bioreactor system to convert wood to potent materials and energy sources with low environmental load and to bioremediate polluted environments. However, the ligninolytic property of these fungi is attributable to many known and unknown enzyme genes, expression of which is inductive, and the factors that determine this expression are not completely understood. The lack of knowledge regarding the ligninolytic property of these fungi is an impediment to the development of a highly effective lignin-degrading fungal strain for the construction of an efficient bioreactor system (Cullen and Kersten 2004). The identification of a master regulator that regulates the entire ligninolytic system in white-rot fungi could be used as a target for breeding a high lignin-degrading strain and for furthering our understanding of the lignin-degradation system in these fungi.

*Phanerochaete chrysosporium*, which is the most widely researched white-rot fungus in the world, has 2 families of lignin-degrading peroxidases designated lignin peroxidase (LiP) and manganese peroxidase (MnP) (Heinzkill and Messner 1997). LiP and MnP are thought to play an important role in initiating the lignin degrading reaction of the fungus, because they can cleave lignin structures extracellularly in the first step of lignin mineralization (Cullen and Kersten 2004; Gold et al. 1984; Tien and Kirk 1984). Moreover, LiP and MnP themselves also have potential applications in treating textile effluent (Sedighi et al. 2009; Singh et al. 2010). However, their expression is inductive, related to unknown factors, and known to be unstable, as is the entire ligninolytic system. Information concerning the LiP and MnP expression system is highly important and requisite not only for better understanding the expression of the entire ligninolytic system, but also for molecular breeding of high LiP- and/or high MnP-producing strains.

MacDonald et al. (1984) reported that intracellular 3'-5'-cyclic adenosine monophosphate (cAMP) levels increased during *P. chrysosporium *degradation of straw lignin to CO_2 _under low nitrogen conditions. Boominathan and Reddy (1992) subsequently indicated that atropine application to *P. chrysosporium *cultures repressed LiP and MnP activity, with decreasing intracellular cAMP levels. However, the relationship between cAMP and LiP and MnP expression remained unclear because the mechanism by which atropine reduced cAMP was not established, and the cAMP reduction may have been caused by repression of the enzymes. Recently, Singh et al. (2011) also reported that cAMP and 3'-isobutyl-1-methylxanthine (IBMX), which is an inhibitor against phosphodiesterase (PDE), increased MnP activity. However, the effect on LiP expression was not mentioned in the report and details of the mechanism, including the effect on LiP and MnP transcriptions and the relationship between cAMP signaling and other signal transduction factors, have yet to be determined.

In this study, we demonstrate that cAMP and IBMX increase the transcription levels of most LiP and MnP isozyme genes. We also investigated the relationship between the cAMP pathway and calmodulin (CaM), which is the major second messenger in the eukaryotic calcium signaling pathway. The CaM gene (*cam*) is present as a single isoform in the *P. chrysosporium *genome (Martinez et al. 2004). We previously revealed that the CaM pathway is required for expression of *lip *and *mnp *genes in *P. chrysosporium *(Minami et al. 2007; Minami et al. 2009; Sakamoto et al. 2010), but the relationship between these signaling factors that leads to LiP and MnP expression has remained unclear. Here, we report experimental results suggesting that CaM expression is regulated by the cAMP pathway, and that cAMP controls LiP and MnP expression mainly through regulation of CaM expression.

## Materials and methods

### Culture conditions

*P. chrysosporium *RP78 (FGSC #9002, ATCC MYA-4764™) (Stewart et al. 2000) was kindly provided by Dr. Gaskell and Dr. Cullen, USDA, Forest Products Laboratory, Madison, WI. Mycelia were maintained at 37°C on yeast malt peptone glucose (YMPG) plates (0.2% w/v yeast extract, 1% w/v malt extract, 0.2% w/v peptone, 1% w/v glucose, 0.1% w/v asparagine, 0.2% w/v KH_2_PO_4_, 0.1% w/v MgSO·H_2_O, 2% w/v agar, and 0.0001% w/v thiamine). Fungal mycelia were inoculated onto the YMPG plates and incubated at 37°C for 6 days to produce conidia. The conidia in culture were harvested in sterilized water, filtered through a 100-μm nylon cell strainer, and washed with sterilized water. The collected conidia (5 × 10^6^) were then inoculated into a 200-ml Erlenmeyer flask under static conditions at 37°C. This flask contained 20 ml nitrogen-limited medium (1% w/v glucose, 20 mM Na-phthalate [pH 4.5], 0.0001% w/v thiamine, 1.2 mM ammonium tartrate, 0.4 mM veratryl alcohol, and 1% v/v Basal III medium [20 g KH_2_PO_4_, 5.3 g MgSO_4_, 1 g CaCl_2_, 50 mg MnSO_4_, 100 mg NaCl, 10 mg FeSO_4_·7H_2_O, 10 mg CoCl_2_, 10 mg ZnSO_4_·7H_2_O, 10 mg CuSO_4_, 1 mg AlK(SO_4_)_2_·12H_2_O, 1 mg H_3_BO_3_, 1 mg Na_2_MoO_4_·2H_2_O, and 150 mg nitrilotriacetate in 1 l ddH_2_O]) (Kirk et al. 1978). After incubation for 48 h under air, 3 mM veratryl alcohol was added as a stabilizer of LiP (Cancel et al. 1993), and the air in the headspace of the flask was replaced with O_2 _gas every 24 h (Kirk and Farrell 1987).

### Chemicals

Adenosine 3'-5'-cyclic monophosphate sodium salt monohydrate (cAMP-NaOH) was purchased from Sigma-Aldrich, Tokyo, Japan. IBMX was purchased from Wako, Osaka, Japan. This drug inhibits PDE and results in high cAMP levels. The typical CaM antagonist N-(6-aminohexyl)-5-chloro-1-naphthalenesulfonamide (W-7) hydrochloride was purchased from Wako, Osaka, Japan. This antagonist binds calcium-loaded CaM to block its Ca^2+ ^signal messenger function (Osawa et al.1998). W-7 repressed all LiPs and MnPs at the transcriptional level via CaM inhibition (Sakamoto et al. 2010).

Dimethyl sulfoxide (DMSO), used as the solvent for IBMX and W-7, was purchased from Nacalai Tesque, Kyoto, Japan. Two days after starting the cultures, 5 mM cAMP, 500 μM IBMX, and 100 μM W-7 were added. DMSO, instead of IBMX or W-7, was added to the culture as a control, which had no effect on enzyme activities and hyphal growth (Sakamoto et al. 2010). The concentration of W-7 is used as in previous report (Sakamoto et al. 2010). The preliminary experiments revealed that 5 mM cAMP or 500 mM IBMX increases LiP and MnP activities significantly, but 1 mM cAMP or 100 mM IBMX not. However, effects of 5 mM cAMP or 500 mM IBMX alone against LiP and MnP activity were not sufficiently reproducible (data not shown). In these experiments, 500 μM IBMX and 5 mM cAMP were added together into cultures, so that the activities were stabilized.

### Determination of ligninolytic enzyme activity

LiP activity was assayed using the method described by Tien and Kirk (1988). The enzyme was incubated with 0.8 mM veratryl alcohol, 100 mM Na-tartrate buffer (pH 3.0), and 250 μM H_2_O_2_. The extinction coefficient of veratryl aldehyde (oxidized veratryl alcohol) at 310 nm is 9,300 M^-1 ^cm^-1^. One unit of enzyme activity represents the oxidation of veratryl alcohol to veratryl aldehyde at a rate of 1 μM/min.

MnP activity was assayed using the method described by Paszczyński et al. (1988). This enzyme was incubated with 0.4 mM guaiacol, 50 mM Na-lactate buffer (pH 4.5), 200 μM MnSO_4_, and 100 μM H_2_O_2_. The extinction coefficient of oxidized guaiacol at 465 nm is 12,100 M^-1 ^cm^-1^. One unit of enzyme activity represents guaiacol oxidation at 1 μM/min. The above assays were repeated 4 times, and the means and standard deviations of enzyme activity were calculated.

### Measurement of dry fungal weight

The culture of each flask was recovered and washed with ddH_2_O on gauze. The water contained within cultures was removed by drying at 105°C for 10 hours, and the weight of fungal bodies was measured.

### Determination of intracellular cAMP level

To confirm the effect of W-7, intracellular cAMP levels under the control and W-7-treated conditions were measured using the Tropix^® ^cAMP-Screen™ chemiluminescent ELISA System (Applied Biosystems, Foster, USA) and PLATE LUMINO (Stratec Biomedical Systems, Birkenfeld, Germany) according to the manufacturers' protocols. For each culture condition, cAMP was extracted with ethanol, which had been previously chilled to -80°C.

### Real-time reverse transcription polymerase chain reaction

**Figure 1:**
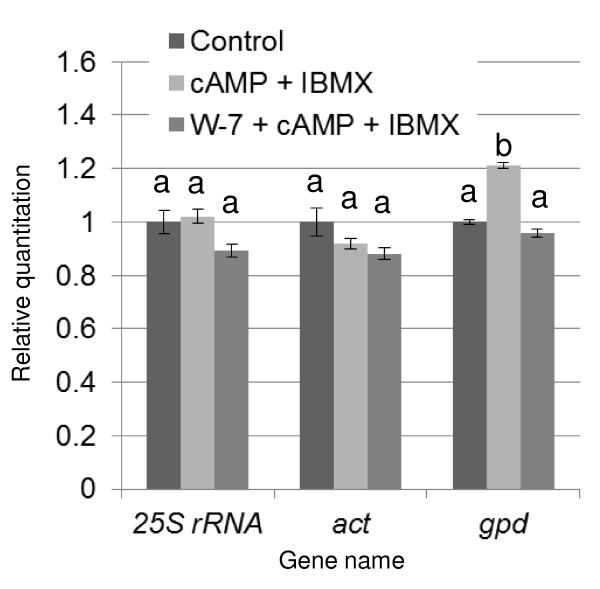
**Relative quantity of transcripts of the 25S rRNA (transcribed by RNA polymerase I), *act *(encoding actin), and *gpd *(encoding GAPDH) genes (transcribed by RNA polymerase II) under various conditions for determination of the internal standard (Figure 4)**. Drugs were added into 48 h culture, and total RNA was extracted from each culture at 24 h after the drug addition. Each real-time RT-PCRs was performed using 3 ng total RNA. Error bars show the SD for 4 biological repetitions. A common letter indicates cases where values were insignificantly different between drug groups (P < 0.05), estimated by Turkey's HSD test following one-way factorial ANOVA. Primers 5'-CGTCAACGACCCCTTCATTG-3' and 5'-CGACATAGAGCTTGCCGTCCT-3' were used for the *gpd *gene. The other primers are listed in Sakamoto et al. (2010).

Quantitative real-time reverse transcription polymerase chain reaction (RT-PCR) analysis was conducted as previously described (Sakamoto et al. 2010). Total RNA was isolated using ISOGEN (Nippon Gene, Tokyo, Japan) according to the manufacturer's protocol. After treatment with RNase-free DNase (TaKaRa, Shiga, Japan), mRNA was reverse transcribed using the PrimeScript RT Regent Kit (TaKaRa, Shiga, Japan) according to the manufacturer's instructions and used for analysis. Quantitative real-time RT-PCR amplification was carried out for all isozyme genes of ligninolytic peroxidase, i.e. 10 *lip *isozyme genes (protein_id 10957, 121822, 131738, 6811, 11110, 122202, 8895, 121806, 131707, 131709), 5 *mnp *isozyme genes (protein_id 140708, 3589, 878, 8191, 4636), and *cam *(protein_id 10767). An actin gene (protein_id 139298) was used as endogenous reference gene, which was not valuable in quantity of its transcript among the culture conditions used in this study (Figure 1). The genes were predicted using data from the *P. chrysosporium *v2.0 genome database (Martinez et al. 2004) available at DOE Joint Genome Institute (JGI; http://genome.jgi-psf.org/Phchr1/Phchr1.home.html). The amplification was performed using gene-specific primers (Sakamoto et al. 2010) and SYBR^® ^Premix Ex TaqTM II (TaKaRa, Shiga, Japan). The experiment was repeated 4 times. PCR amplifications using a Thermal Cycler Dice TM real-time system (TaKaRa, Shiga, Japan) were performed as follows: (i) an initial denaturation step at 95°C for 10 s and (ii) 40 cycles, with each cycle consisting of denaturation at 95°C for 5 s and annealing and elongation at 60°C for 30 s. The standard curve of each gene was constructed from real-time PCR results using dilution series of the PCR product made by the same primer pair template as for real-time RT-PCR. Transcription of each gene was quantified using the standard curve. For comparisons between different culture conditions, the total amount of complementary DNA (cDNA) was normalized against that of actin.

### Statistical analysis

Data were analyzed by one-way factorial, 2-way factorial, or 2-way repeated-measures ANOVA, and significant differences between the groups were determined by Turkey's HSD test or Bonferroni method (*P *< 0.05) using SPSS version 18.01, SPSS Inc.

## Results

### Effect of exogenous cAMP and IBMX on enzyme activity

**Figure 2:**
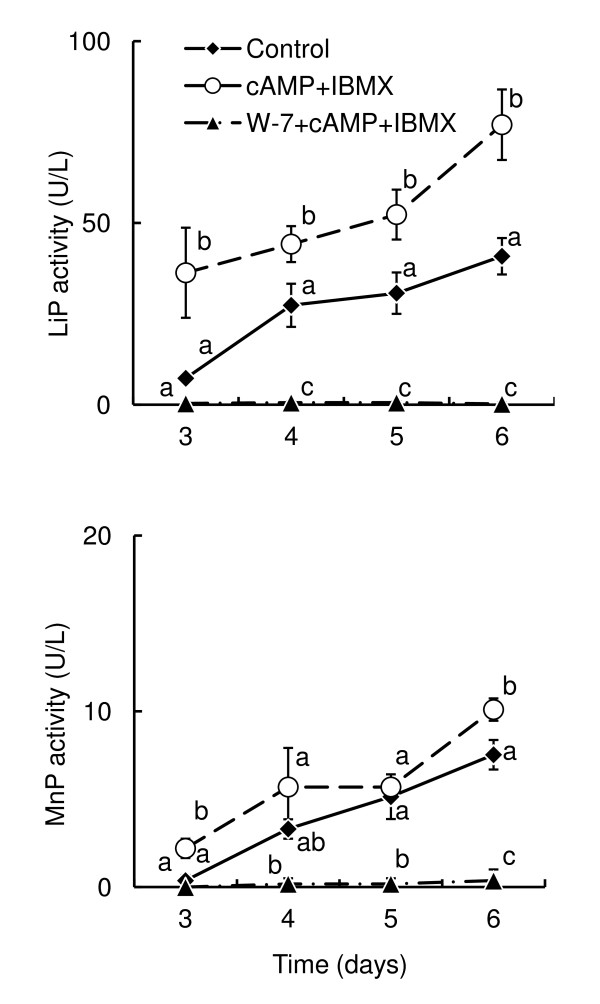
**Time courses of LiP and MnP activity levels in *P. chrysosporium *culture in the presence of various drugs**. Each chemical was added after 48 h incubation. Effect on LiP activity (top panel) and MnP activity (bottom panel) under each condition. Error bars show the standard deviation (SD) for 3 biological repetitions. Mean values not sharing a common letter are significantly different between drug groups on the same day (*P *< 0.05), as estimated by Bonferroni method following 2-way repeated-measures ANOVA.

**Figure 3:**
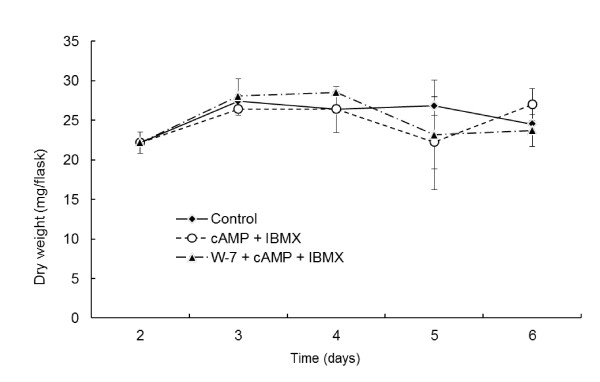
**Time courses of *P. chrysosporium *culture dry weights with various drugs**. Error bars show the SD for 3 biological repetitions. No significant difference was observed with 2-way factorial ANOVA. *P *value of the estimate for the drug groups is more than 0.795. *P *value of the estimate for the 2-factor interaction between drug groups and culture days is more than 0.226.

Time courses of LiP and MnP activity levels were measured following addition of various supplements to *P. chrysosporium *culture at 48 h after culture initiation, at which time their activity was still undetectable. LiP and MnP activity levels statistically significantly increased in the presence of 5 mM cAMP and 100 μM IBMX compared to the no-supplement control (Figure 2). W-7, a CaM inhibitor that repressed the activity and the transcription of the all isozyme genes and did not affect fungal growth in our previous study (Sakamoto et al. 2010), blocked not only the basal activity levels but also the effect of cAMP and IBMX (Figure 2). No significant treatment-related change in hyphal growth (dry weight) of the fungus was observed over the time courses (Figure 3). In the case of addition of only W-7, the result was same as in the case of addition of cAMP, IBMX and W-7 (data not shown), which was already reported by Sakamoto et al. (2010). These results suggest that the cAMP pathway has a positive effect on LiP and MnP expression that can be blocked by CaM inhibition.

### Transcriptions of the isozyme genes following exposure to the stimuli

The genome of *P. chrysosporium *RP78 is predicted to contain 10 and 5 genes encoding LiP and MnP, respectively, using the *P. chrysosporium *v2.0 genome database (Martinez et al. 2004). Real-time RT-PCR was carried out to analyze changes in the quantity of transcription of these genes induced by treatment with various supplements. Total RNA was extracted from the cultures 24 h after addition of supplements at 48 h in culture.

**Figure 4:**
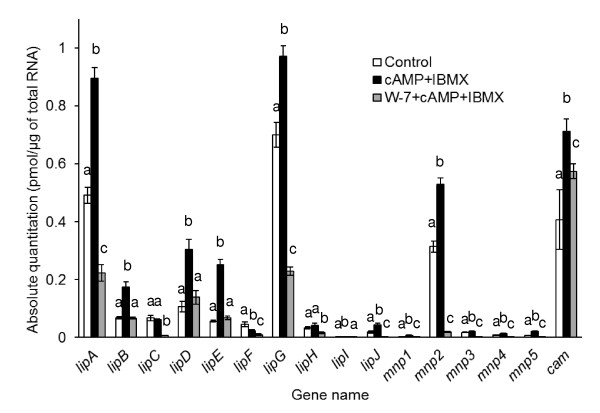
**Absolute quantities of the *lip, mnp*, and *cam *gene transcripts**. Each drug was added after 48 h incubation, and mRNA was extracted from the fungus after 72 h (according to Methods). Error bars show the SD for 4 experimental repetitions. Mean values not sharing a common letter are significantly different between drug groups (P < 0.05), estimated by Turkey's HSD test following one-way factorial ANOVA. This figure shows the representative result of same experiments. A same result was obtained when same experiment was biologically repeated (data not shown).

Transcript for most of these isozyme genes was statistically significantly increased in the presence of cAMP and IBMX compared to the no-supplement condition. Notably, transcripts of all the major isozymes (*lipA, lipG*, and *mnp2*), which we observed to be expressed more highly than the other genes, significantly increased. Only expression of *lipF *was repressed in this condition (Figure 4). This finding suggests that the transcription of most isozymes can be increased by exogenously stimulated cAMP signaling, which likely at least partially led to the increase in LiP and MnP activity. W-7 functioned not only to offset the increase but to decrease gene expression levels of some isozymes, including the major isozymes, to below basal levels in (Figure 4).

The transcription of *cam *was also analyzed. It was upregulated by treatment with cAMP and IBMX, and this effect was partially blocked by W-7.

### Intracellular concentration of cAMP following exposure to W-7

**Figure 5:**
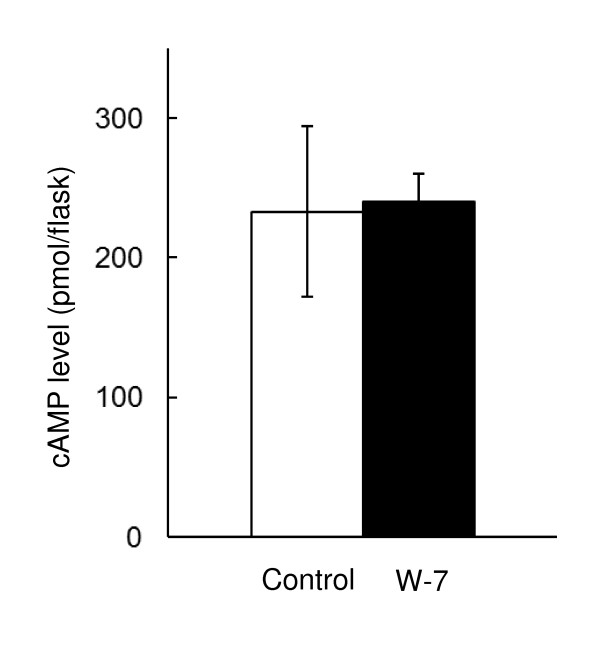
**Effect of W-7 addition on the level of intracellular cAMP of *P. chrysosporium***. Chemicals were added after 48 h culture, and cAMP was eluted from the fungus after 72 h. Error bars show the SD for 3 biological repetitions. No significant difference was observed by *t *test. *P *value is more than 0.826.

As mentioned above, W-7 repressed the activity of LiP and MnP and transcription of *lip *and *mnp *genes even in the presence of cAMP and IBMX, which upregulated transcription of *cam *as well as *lip *and *mnp *genes. Because W-7 can inhibit cAMP signaling, CaM likely acts downstream from cAMP. However, a shortage of cAMP, arising from inhibition of intracellular cAMP production via CaM inhibition, may also possibly result in reducing transcription of the isozyme genes. To clarify this ambiguity, the effect of W-7 on cAMP production was analyzed. Intracellular cAMP concentration following W-7 addition did not change compared to that of control (Figure 5). These results indicate that CaM does not regulate cAMP production, suggesting that the increased cAMP concentration affects the transcription of genes encoding LiPs and MnPs via regulation of CaM transcription.

## Discussion

Expression of all *lip *and *mnp *isozyme genes except *lipC, lipF, lipH *was statistically significantly increased compared to the control condition with the absence of drugs (Figure 4). This finding strongly suggests that cAMP signaling increases *lip *and *mnp *transcription levels. We have also previously reported that CaM transcription was repressed following exposure to atropine (Minami et al. 2009), and that *lip *and *mnp *isozyme gene transcripts were downregulated by addition of the CaM inhibitor, W-7 (Sakamoto et al. 2010). These observations indicated that atropine decreased endogenous cAMP concentration, which resulted in insufficient cAMP signaling to induce upregulation of *cam *gene transcription. This evidence is strongly supported by the observation that *cam *gene transcription was also increased by the addition of cAMP and IBMX (Figure 4). Moreover, W-7 blocked the transcription of *lip *and *mnp *isozymes in the presence of cAMP and IBMX (Figure 4) and did not affect intracellular cAMP concentration (Figure 5). All these data suggest that cAMP signaling increases LiP and MnP transcripts through the induction of *cam *transcription.

**Figure 6:**
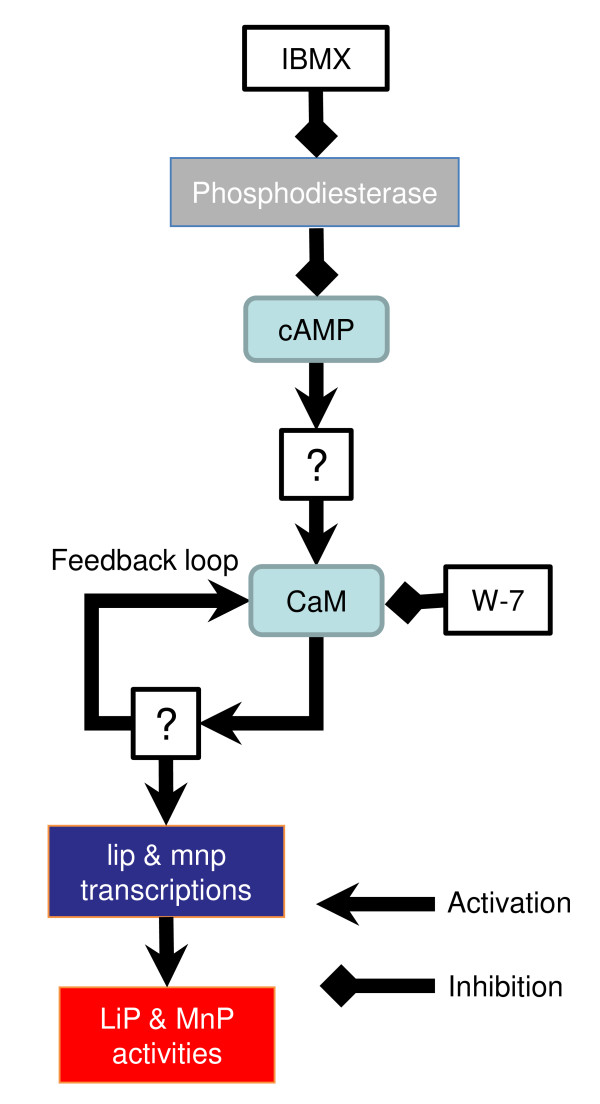
**Model of the predicted cAMP and CaM signaling pathways for the production of LiPs and MnPs in *P. chrysosporium***.

Nevertheless, CaM function may not be the only factor to induce transcription of *lip *and *mnp *genes, because W-7 did not seem to completely block transcription of *lip *isozyme genes (Figure 4) although it repressed almost all LiP activity (Figure 2). To some extent, W-7 also blocked the *cam *transcription induced by cAMP and IBMX (Figure 4), suggesting the existence of a CaM signaling feedback loop that comprises a self-inducible system in which CaM protein itself upregulates *cam *expression as discussed in our previous report (Sakamoto et al. 2010). Further study is required to determine whether the CaM has other functions including post-transcriptional effects on the expression of LiP and MnP. Additionally, *lipF *regulation, transcription of which was not upregulated following exposure to cAMP and IBMX, should also be further analyzed. The diagram of cAMP and CaM pathways for the LiP and MnP expression has been updated based on the present results (Figure 6). Of course, there are many other regulating factors, which are not described in Figure 6, for example, Mn^2+ ^that causes reverse effect between LiP and MnP production (Bonnarme 1990) and nitrogen starvation and reactive oxygen species (ROS) as described below.

*P. chrysosporium *must be starved of nitrogen or carbon and exposed to ROS to induce expression of LiP and MnP at the transcriptional level (Belinky et al. 2003; Li et al. 1995). cAMP was reported to correlate with starvation conditions regardless of ROS (Belinky et al. 2003), and another Ca^2+ ^signaling factor, protein kinase C, was reported to demonstrate involvement in ROS signaling underlying LiP expression (Matityahu et al. 2010). However, our results indicate cross-talk between the cAMP and Ca^2+ ^signaling pathways. Although cAMP signaling may activate the downstream signaling pathway and ultimately induce LiP and MnP expression in the presence of ROS, cAMP signaling pathway genes are not good breeding targets, because cAMP signaling is important not only to expression of LiP and MnP but also to various functions of fungi involved in vegetative growth (Kronstad et al. 1998; Liebmann et al. 2003; Takano et al. 2001). The same goes for CaM, which is necessary for hyphal growth and many physiological functions of fungi (Ahn and Suh 2007; Davis et al. 1986; Rao et al. 1998; Sato et al. 2004; Wang et al. 2006). Although the addition of 100 μM W-7 at 2 days after culture initiation did not significantly affect fungal growth using our method (Figure 3), 200 μM W-7 decreased fungal growth using the same method (Sakamoto et al. 2010). We are currently investigating CaM-interacting proteins to analyze the downstream pathway regulated by CaM with the aim to identify a breeding target that does not affect fungal growth, and trying to develop an efficient practicable transformation system of *P. chrysosporium *so that a high throughput detection system for the target gene could be constructed.

The relationship between ROS and CaM still remains to be analyzed. CaM antagonists such as W-7 have been reported to reduce oxidative stress-induced cell death generated by mitochondrial dysfunction in neurons (Lee et al. 2005; Shen et al. 2001). Since the cell death was caused by oxidized cholesterols and, in *Caenorhabditis elegans *and brain of worker honeybees, oxysterol-binding protein-like protein was detected as a protein interacting with CaM (Shen et al. 2008; Calábria et al. 2008), oxysterol produced by ROS may be speculated to interact with a CaM-oxysterol binding protein complex to signal the expression LiP and MnP in *P. chrysosporium*. We will analyze possible correlations following the search for CaM-interacting proteins.
